# Supplementary material for: Enhancing in vivo cell and tissue targeting by modulation of polymer nanoparticles and macrophage decoys
Source: Nat Commun. 2024 May 18;15:4247. doi: 10.1038/s41467-024-48442-7 (PMC11102454; doi:10.1038/s41467-024-48442-7)
Supplement: Supplementary file 1 — Supplementary Information [file 41467_2024_48442_MOESM1_ESM.pdf]

Supplementary Information

Enhancing in vivo cell and tissue targeting by modulation of polymer nanoparticles and macrophage decoys

Authors: Alexandra S. Piotrowski-Daspit[1,2,3‡*], Laura G. Bracaglia[1,4‡*], David A. Eaton[1], Owen Richfield[1], Thomas C. Binns[1,5], Claire Albert[1], Jared Gould[1], Ryland D. Mortlock[1], Marie E. Egan[6,7], Jordan S. Pober[7,8], and W. Mark Saltzman[1, 7, 9, 10*]

Affiliations:
[1]Department of Biomedical Engineering, Yale University, New Haven, CT 06511
[2]Department of Biomedical Engineering, University of Michigan, Ann Arbor, MI 48109
[3]Department of Internal Medicine – Pulmonary and Critical Care Medicine Division, Michigan Medicine, University of Michigan, Ann Arbor, MI 48109
[4]Department of Chemical and Biological Engineering, Villanova University, Villanova, PA 19085
[5]Department of Laboratory Medicine, Yale School of Medicine, New Haven, CT 06510
[6]Department of Pediatrics, Yale School of Medicine, New Haven, CT 06510
[7]Department of Cellular & Molecular Physiology, Yale School of Medicine, New Haven, CT 06510
[8]Department of Immunobiology, Yale School of Medicine, New Haven, CT 06510
[9]Department of Dermatology, Yale School of Medicine, New Haven, CT 06510
[10]Department of Chemical & Environmental Engineering, Yale University, New Haven, CT 06511

‡These authors contributed equally to this work.

(*) Address correspondence to W.M.S., A.S.P., and L.G.B.:

W. Mark Saltzman
Malone Engineering Center 413
55 Prospect Street
New Haven, CT 06511
Phone: (203) 432-3281
Fax: (203) 432-0030
E-mail: mark.saltzman@yale.edu

Alexandra S. Piotrowski-Daspit
NCRC B520 2355
1600 Huron Parkway
Ann Arbor, MI 48109
Phone: (734) 647-2605
E-mail: asapd@umich.edu

Laura G. Bracaglia
White Hall Rm 315
800 Lancaster Avenue
Villanova, PA 19085
Phone: (610) 519-7106
E-mail: laura.bracaglia@villanova.edu

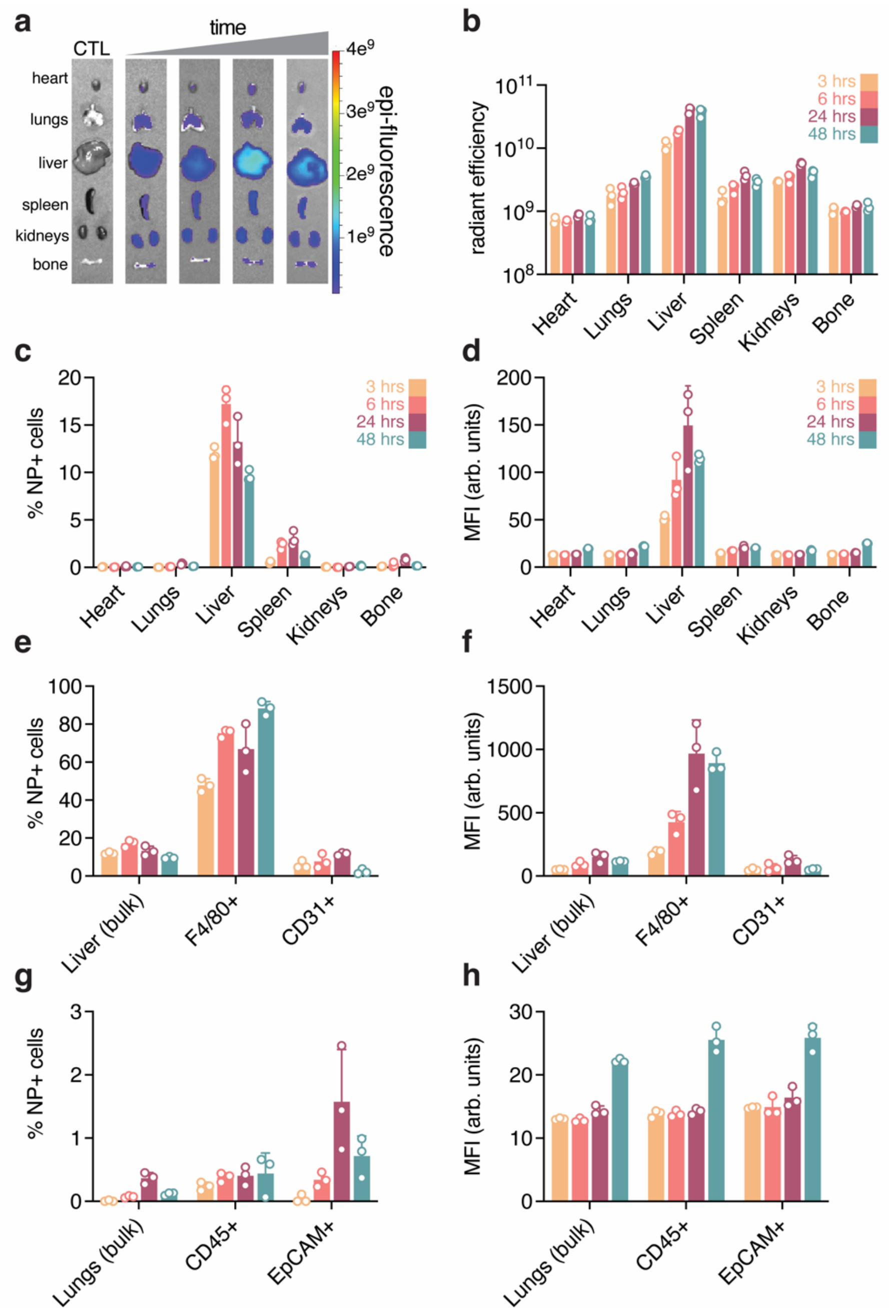

Supplementary Figure 1. Time course of PACE-PEG NP biodistribution. (a) IVIS analysis of PACE-PEG NP uptake in various organs (heart, lungs, liver, spleen, kidneys, and bone) over time (0.5 mg dose per animal, 3 hours: orange, 6 hours: peach, 24 hours: purple, 48 hours: teal). End-point analysis of (b) whole organ fluorescence quantification of PACE-PEG NP uptake in various organs (n = 3 mice per group per organ; error bars represent standard deviation (SD)). End-point analyses of (c) %NP+ cells and (d) mean fluorescence intensity (MFI) in arbitrary units (arb. units) in homogenized organs by flow cytometry (n = 3 mice per group per organ; error bars represent SD). End-point analysis of (e) %NP+ cells and (f) MFI in homogenized liver populations (bulk, F4/80+, and CD31+) by flow cytometry (n = 3 mice per group per population; error bars represent SD). End-point analyses of (g) %NP+ cells and (h) MFI in homogenized lung populations (bulk, CD45+, and EpCAM+) by flow cytometry (n = 3 mice per group per population; error bars represent SD).

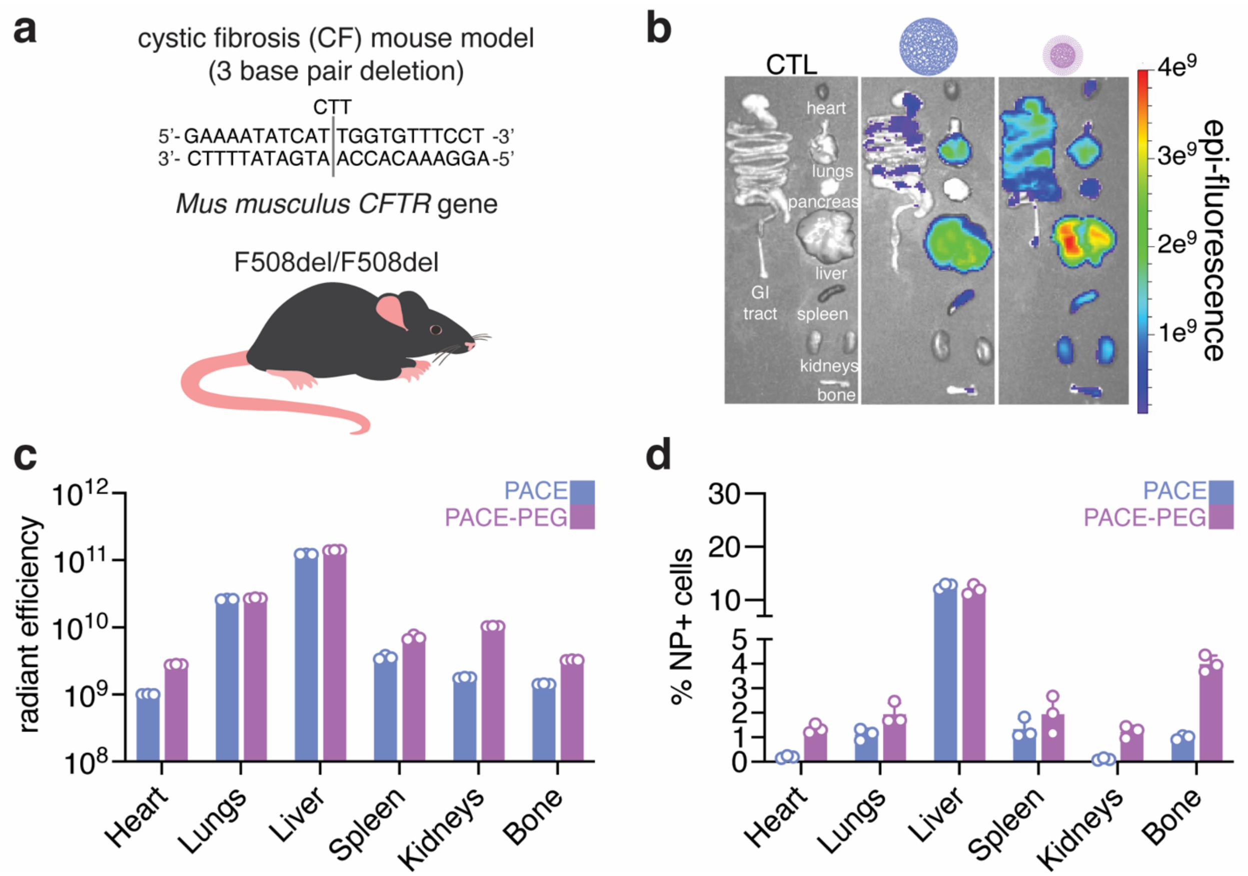

Supplementary Figure 2. PACE and PACE-PEG NP biodistribution in the F508del CF mouse model. (a) Schematic of the F508del CF mouse model illustrating the CF-associated 3 bp deletion in the CFTR gene. (b) Representative end-point IVIS analysis of PACE NP (blue) and PACE-PEG NP (purple) uptake in various organs (heart, lungs, pancreas, liver, spleen, kidneys, bone, and gastrointestinal (GI) tract)). End-point analyses of (c) whole organ fluorescence quantification of PACE and PACE-PEG NP uptake in various organs (n = 3 mice per group per organ; error bars represent standard error of the mean (SEM)), (d) %NP+ cells in homogenized organs by flow cytometry (n = 3 mice per group per organ; error bars represent SEM).

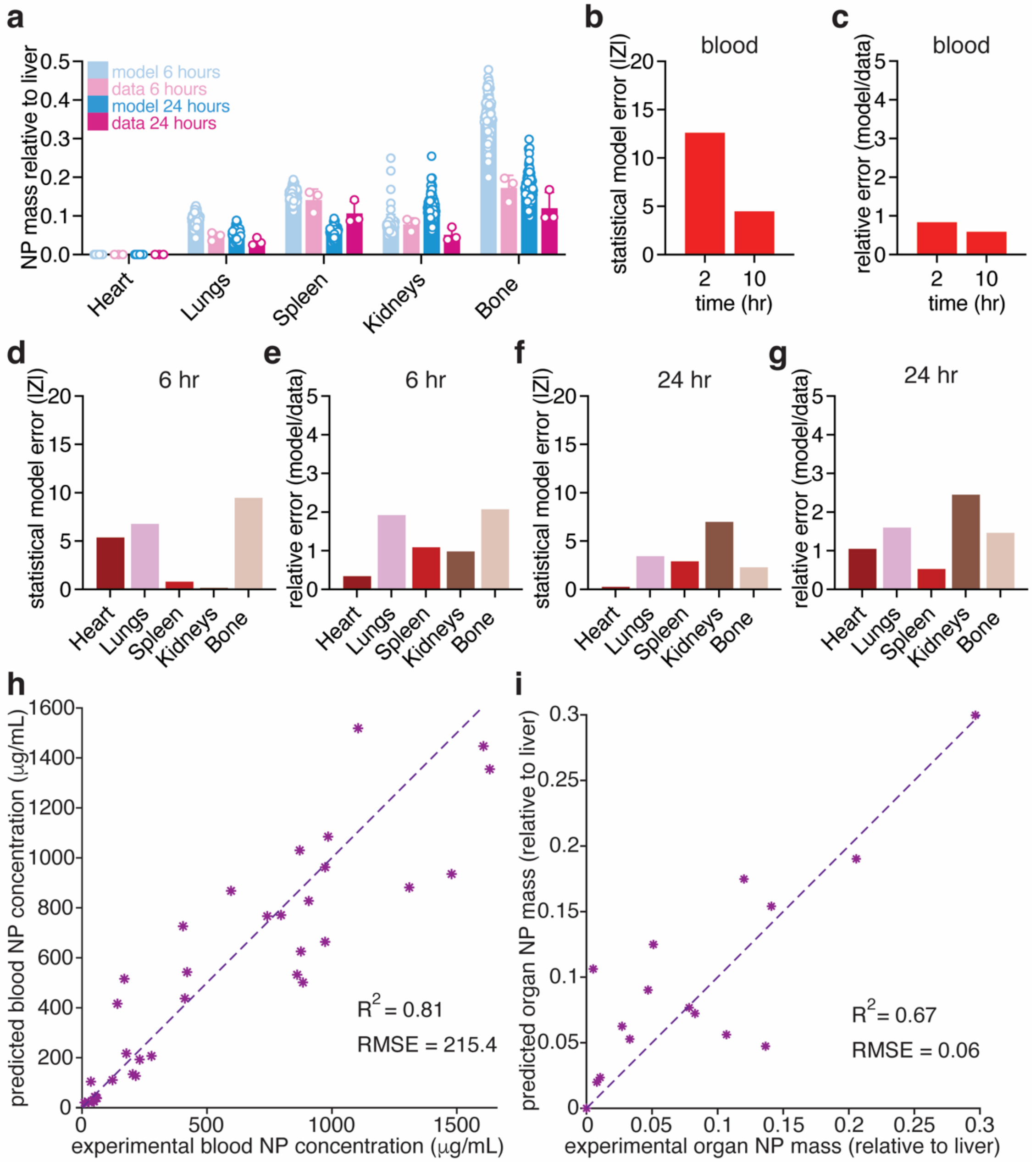

Supplementary Figure 3. PBPK model validation. Experimental results of PACE NPs in the organs are compared to model results at the time points for which the data was not used to parameterize the PBPK model of PACE-PEG biodistribution in mice, shown as the organ NP mass (relative to the liver) at 6 and 24 hours post administration. (a) Mean values of the model (blue) and data (pink) are shown in blue and pink, respectively, with error bars to indicate standard deviations (model: n = 100 simulations per group per organ; data: n = 3 mice per group per organ). (b,c) The Z value caluclated based on the model and the data for the blood (b) and the corresponding model error quantified as the relative value of the model mean as compared to the data mean for the blood (c). These metrics are also shown for the organs at 6 hours post administration (d,e) and and 24 hours post administration (f,g). (h) Actual and model-predicted PACE-PEG NP blood concentration at all time points for all doses are compared, with calculated R^2 and root mean squared error (RMSE). (i) Organ mass of NPs normalized to the liver at all time points (3, 6, 24 and 48 hours post-administration) are compared, with calculated R^2 and RMSE.

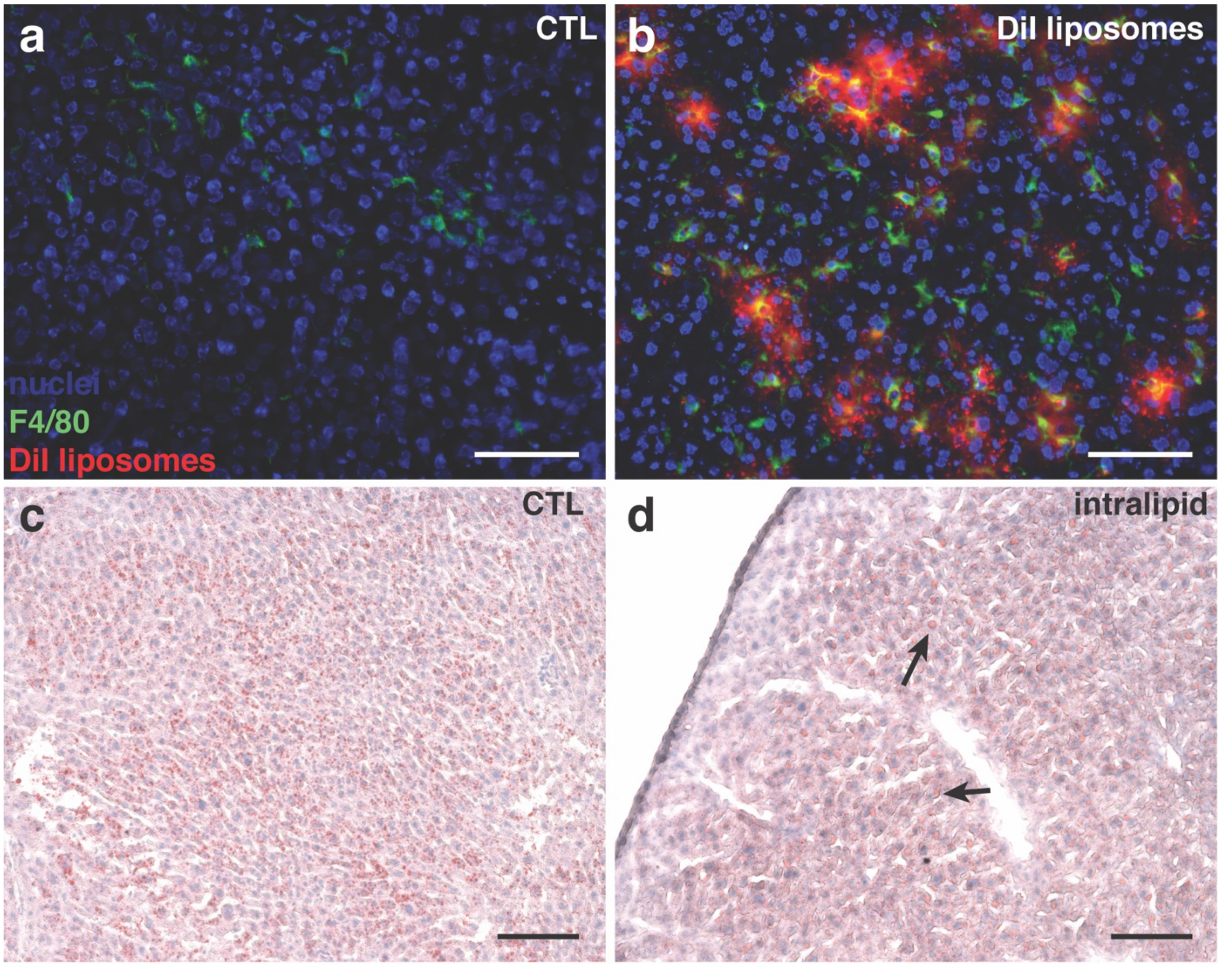

Supplementary Figure 4. Liver occupation by liposomes and intralipid. Representative epifluorescence liver images from three independent experiments of (**a**) untreated control and (**b**) DiI-loaded liposome-treated animals 24 hours post-IV administration. Nuclei are shown in blue, F4/80^{+} macrophages are shown in green, and DiI liposomes are shown in red. Scale bars, 100 μm. Representative liver histology images from three independent experiments with oil red "O" staining (red) of (**c**) untreated control and (**d**) intralipid-treated animals 24 hours post-IV administration. Arrows indicate lipid staining. Scale bars, 100 μm.

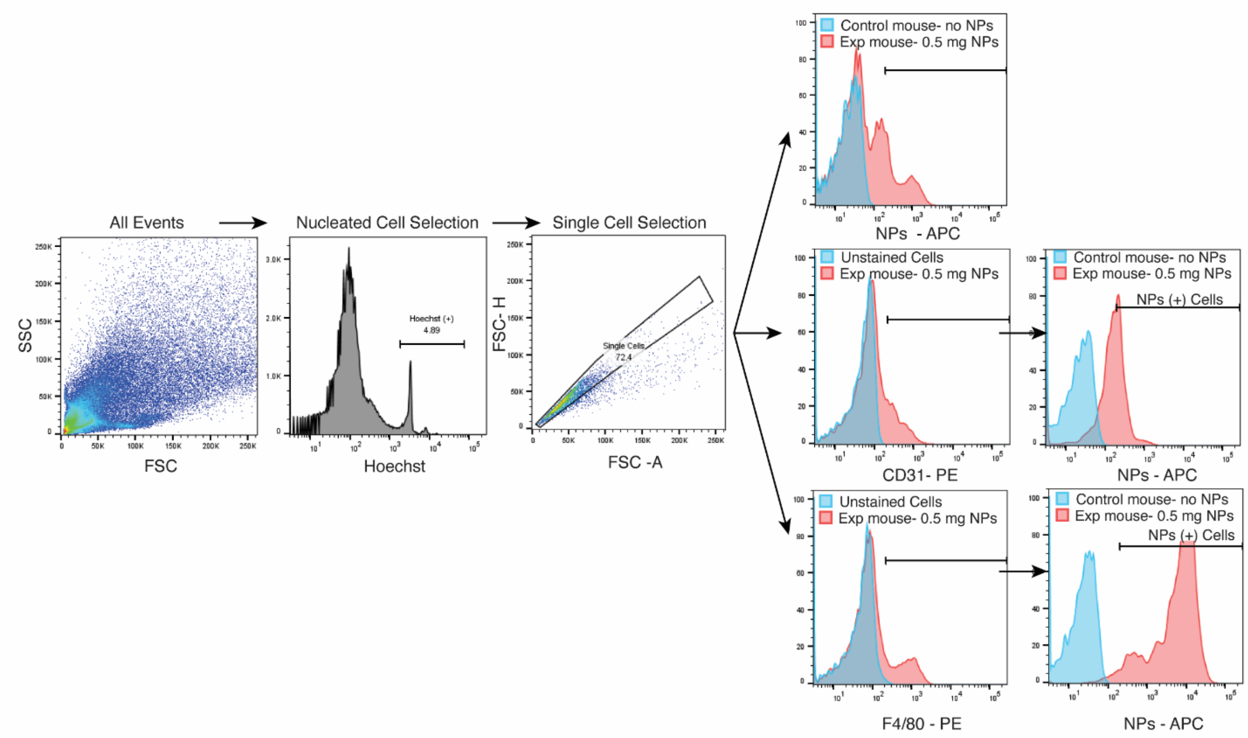

Supplementary Figure 5. Gating Strategy for Homogenized Organs Analyzed by Flow Cytometry. Cells were first selected from total events using a Hoechst 33342 stain to remove non-nucleated cells and fragments. Nucleated cells were then selected for single cells using a forward scatter height by forward scatter area selection. From there, bulk cells were evaluated for NP signal using the fluorescent dye contained within the NP (DiD-APC channel) and compared to cells from animals without NPs. Bulk cells were also sorted into specific cell populations, using fluorescent antibody markers, and gated based on unstained cells from the same population. These specific cell types were then evaluated for NP signal using the fluorescent dye contained within the NP (DiD-APC channel), and compared to cells from animals without NPs.

Supplementary Table 1. Characterization data for DiD and siRNA PACE NP formulations. DCM, dichloromethane; EtAc, ethyl acetate.

Material	Solvent	Diameter (nm)	Polydispersity Index (PDI)	Zeta Potential (mV)
PACE 60% PDL (DiD)	DCM	230	0.17	25.4
PACE 60% PDL (DiD)	50:50 DCM:EtAc	200	0.11	22.6
PACE 60% PDL (DiD)	50:50 DCM:EtAc	140	0.08	12
PACE 60% PDL COOH (DiD)	DCM	240	0.16	17.2
PACE 60% PDL COOH (DiD)	50:50 DCM:EtAc	190	0.24	18.9
PACE 60% PDL COOH (DiD)	50:50 DCM:EtAc	140	0.18	15.9
PACE 60% PDL PEG (DiD)	Chloroform	180	0.22	28.4
PACE 60% PDL PEG (DiD)	50:50 Chloroform:EtAc	160	0.14	33.9
PACE 60% PDL (PCSK9 siRNA)	DCM	237	0.18	25.9

Supplementary Table 2. Physiological parameters used in mathematically modeling PACE NP pharmacokinetics.

Parameter	Description	Value	Units	Reference
BW	Body weight	0.02	kg	1
QC	Cardiac output	16.5	L/h/kg	1
QHC	Heart blood flow fraction	1	ND	1
QKC	Kidney blood flow fraction	0.091	ND	1
QLC	Liver blood flow fraction	0.161	ND	1
QSC	Spleen blood flow fraction	0.011	ND	1
QLuC	Lung blood flow fraction	1	ND	1
QBoneC	Bone blood flow fraction	0.072	ND	2
QBRC	Brain blood flow fraction	0.033	ND	1
QRestC	Body blood flow fraction	0.632	ND	1
VHC	Heart volume fraction	0.005	ND	3
VKC	Kidney volume fraction	0.017	ND	1
VLC	Liver volume fraction	0.055	ND	1
VSC	Spleen volume fraction	0.005	ND	1
VLuC	Lung volume fraction	0.007	ND	1
VBoneC	Bone volume fraction	0.011	ND	4
VBRC	Brain volume fraction	0.017	ND	1
VRestC	Body volume fraction	0.834	ND	1
VBloodC	Blood volume fraction	0.049	ND	1
VVenC	Venous blood volume fraction	0.039	ND	1
VArtC	Arterial blood volume fraction	0.010	ND	1
BVH	Heart blood fraction	0.240	ND	1*
BVK	Kidney blood fraction	0.240	ND	1
BVL	Liver blood fraction	0.310	ND	1
BVS	Spleen blood fraction	0.170	ND	1
BVLu	Lung blood fraction	0.500	ND	1

BVBone	Bone blood fraction	0.110	ND	4
BVBR	Brain blood fraction	0.030	ND	1
BVRest	Body blood fraction	0.040	ND	1
CNH	Heart cell number	3.3×10^4	Cells	5
CNK	Kidney cell number	142.0×10^6	Cells	6
CNL	Liver cell number	264.8×10^6	Cells	6
CNS	Spleen cell number	193.9×10^6	Cells	6
CNLu	Lung cell number	86.1×10^6	Cells	6
CNBone	Bone cell number	300.0×10^6	Cells	4

*In lieu of a definitive value of vascularization of the heart, we assume it is the same as the kidney.

Supplementary Table 3: Parameters of the mathematical model. Parameters are presented in terms of their mean and standard deviation (SD). The coefficient of variation (CV) and normalized sensitivity coefficient (NSC) are presented for each parameter. Gray boxes include 'not applicable' (N/A) and CVs corresponding to parameters that were assumed correlated to estimated parameters. Blue boxes indicate significant CV and/or NSCs. 'PEG/Au' refers to the value of that parameter corresponding to the source material, in which PEGylated gold NPs were modeled.[1]

Parameter	Description		PEG/Au	Mean	SD	Units	CV	NSC
PA_{Heart}	Vascular permeability	Heart	N/A	8.4e-11	2.1e-11	L/hr	0.3	0
PA_{Kidney}		Kidney	1.0e-3	1.0e-6	0	L/hr	N/A	N/A
PA_{Liver}		Liver	1.0e-3	1.1e-3	1.2e-4	L/hr	0.1	-0.04
PA_{Spleen}		Spleen	1.0e-3	2.0e-4	8.2e-5	L/hr	0.4	0
PA_{Lung}		Lung	1.0e-3	2.5e-6	8.9e-7	L/hr	0.4	0
PA_{Bone}		Bone	N/A	10.2e-6	2.7e-6	L/hr	0.3	0
PA_{Brain}		Brain	1.0e-6	1.8e-10	7.8e-11	L/hr	0.4	N/A
PA_{Body}		Body	1.0e-6	1.8e-10	7.8e-11	L/hr	0.4	0
$(K_{max})_{Liver}$	Maximum phagocytosis	Liver	4.0	1.5e-2	4.5e-3	1/hr	0.3	-0.04
$(K_{max})_{Spleen}$		Spleen	10.0	2.6e-1	1.1e-1	1/hr	0.4	0
$(K_{max})_{Lung}$		Lungs	0.10	1.1e-3	4.7e-4	1/hr	0.4	0
$(K_{max})_{Kidneys}$		Kidneys	0.10	3.3	1.5	1/hr	0.5	0
K_{bile}	Excre -tion	Liver	1.2e-3	1.1e-3	1.2e-4	1/hr	0.1	-0.6
K_{urine}		Kidneys	1.2e-4	0	0	1/hr	N/A	N/A
$(K_{rel})_{Liver}$	Phagocytic Cell NP	Liver	7.5e-3	1.5e-4	4.5e-5	1/hr	0.3	0
$(K_{rel})_{Spleen}$		Spleen	3.0e-3	2.6	1.14	1/hr	0.4	0
$(K_{rel})_{Lungs}$		Lungs	5.0e-3	7.3e-3	2.9e-4	1/hr	0.4	0
$(K_{rel})_{Kidneys}$		Kidneys	1.0e-2	3.3	1.5	1/hr	0.5	0
$(K_{50})_{Liver}$	Time to half-maximum	Liver	24.0	0.5	0.2	hr	0.5	0
$(K_{50})_{Spleen}$		Spleen	24.0	0.9	0.4	hr	0.4	0
$(K_{50})_{Lungs}$		Lungs	24.0	17.2	7.9	hr	0.4	0
$(K_{50})_{Kidneys}$		Kidneys	24.0	125.4	43.9	hr	0.5	0
n_H	Hill coefficient		0.10	2.8	1.3	ND	0.5	0

Supplementary Methods

PBPK Model Parameterization

A constant rate of administration was assumed to last for the first 20 seconds of the simulation, with a dose rate calibrated to ensure the correct dose was administered. We initially set elimination constants $K_{urine} = K_{bile} = 0$ and verified that the administered dose (0.5 mg of PACE-PEG NPs, as in experiments in Figure 1) was conserved. We then assumed that $K_{urine} = 0$, and $PA_{Kidney} = 0$, based on previous experiments administering similarly sized polymeric NPs to *ex vivo* perfused human kidneys[7]. In these experiments, practically no NPs were excreted in the urine or diffused into the tubular or interstitial spaces. These experiments showed a far greater NP uptake in glomeruli than peritubular capillaries, which motivated our assumption that all NP uptake in the mouse kidneys in our study (an almost negligible amount, as shown in figure 1) is exclusively due to mesangial cell uptake of NPs in the glomeruli. Our second assumption was that the brain vascular permeability (denoted PA_{Brain}) was the same as the vascular permeability of the 'body' compartment. Mouse brain uptake of PACE NPs is practically negligible[8], such that it was not required to quantify the NP concentration in brain tissue or include a detailed description of NP distribution in the brain compartment in our model. Lastly, we assumed that P was the same across gold and PACE-PEG NPs for all organs, and that the heart and bone marrow had the same P as the non-liver organs.

As stated previously, the model used in our study was originally developed using biodistribution data from PEGylated gold NPs in mice. To fit the model to our PACE NP data, we re-parameterized the model by modifying the gold NP model's parameter values using Monte Carlo Importance Sampling (MCIS)[9]. MCIS involves running serial (10^4) simulations with the model, sampling parameter values from prescribed probability distributions, and updating the parameter distributions based on how well the simulation results fit the data. At the end of MCIS, parameters are represented as probability distributions, such that subsequent model simulations can be generated by sampling these parameter distributions to generate model results that are also probabilistic in nature. As such, the model not only provides a predicted value of NP concentration in the blood and tissue compartments, but also provides the probability that the model result lies within any specified interval. Beyond the utility of modeling PACE NP pharmacokinetics probabilistically, MCIS allows for the uncertainty of the experimental data to be encapsulated within the model, which can be used to avoid erroneous predictions that are not properly backed by the experimental data.

We parameterized the model with MCIS using a three-step procedure. Firstly, we used MCIS to rescale the parameters used to model PEGylated gold NPs by probabilistically sampling from uniform distributions to determine the order of magnitude difference required to match the model to our PACE biodistribution data. Once we rescaled the parameters, we then performed another round of MCIS, sampling from tighter uniform distributions to finetune the parameter distributions. Lastly, once the parameters were of the proper scale, we performed a third round of MCIS sampling from normal distributions. With each step, the model result was compared with experimental data in order to determine how best to rescale and ultimately estimate the parameter distributions. The data used to parameterize the model included the PACE-PEG NP concentration in the blood at 0.03, 1, 8 and 48 hours, and the relative NP concentration in each non-liver organ

at 3 and 48 hours (excluding the 'body' and brain compartments), normalized to the liver (endpoint biodistribution data).

Biodistribution data was measured by quantifying NP fluorescence, which does not translate directly to the model-predicted mass of NPs in each organ. To compare the model results to the experimental data, we had to normalize the experimental data; the flow cytometry-based MFI for each organ (Figure 2f) was multiplied by that organ's cell number (Supplementary Table 2) and divided by the corresponding value (the product of MFI and cell number) of the liver:

$$R_i^{Liver} = \frac{MFI_i CN_i}{MFI_{Liver} CN_{Liver}}, \tag{1}$$

For the subscript i pertaining to each of the five non-liver organs. This resulted in five ratios R_i^{Liver} of fluorescence intensity corresponding to the five non-liver organs that were evaluated in our experiments. The experimental data R_i^{Liver} was calculated for each organ in each mouse, such that R_i^{Liver} is distributed as a Gaussian with mean and standard deviation. The model results were compared to these experimental values by dividing the model-predicted sum of the mass of NPs in non-vascular sub-compartments in each organ by the sum of the NP mass in the non-vascular sub-compartments of the liver:

$$r_i^{Liver} = \frac{M_{extra}^i + M_{phago}^i}{M_{extra}^{Liver} + M_{phago}^{Liver}} \tag{2}$$

We assume that the mass of NPs in the vascular space of each organ is negligible, after a cardiac perfusion was used to clear the mouse vasculature of unbound NPs. Thus, the vascular NP mass is not used in comparing the model result to the experimental data. For each model simulation, a value of r_i^{Liver} was calculated for each of the five non-liver organs. If the simulation r_i^{Liver} lay within the 99% confidence interval of the experimental data (R_i^{Liver}) for that organ, then that condition was assumed to be met (five possible conditions, at 3 and 48 hours for a total of ten conditions).

To fit the model to the 15 conditions (10 biodistribution and 5 blood concentration), we estimated values for 15 parameters: K_{max} and K_{50} for each of the phagocytic organs (lungs, kidneys, liver and spleen), the Hill coefficient n_H, assumed equal for all phagocytic organs, K_{bile}, and PA for the non-liver organs, excluding the kidney and including the 'body' compartment. We estimated these parameter values (subscript PACE) by scaling the parameter values associated with PEGylated gold NPs (subscript Au):

$$(K_{max})_{Liver,PACE} = 10^{u_{41}} \tag{3}$$

$$(K_{max})_{Kidneys,PACE} = 10^{u_{41}} \tag{4}$$

$$(K_{max})_{Lungs,PACE} = 10^{u_{41}} \tag{5}$$

$$(K_{max})_{Spleen,PACE} = 10^{u_{41}} \tag{6}$$

$$(K_{bile})_{PACE} = (K_{bile})_{Au} \times 10^{u_{20}} \tag{7}$$

$$(n_H)_{PACE} = (n_H)_{Au} \times 10^{u_{01}} \tag{8}$$

$$(K_{50})_{Liver,PACE} = (K_{50})_{Liver,Au} \times 10^{u_{21}} \tag{9}$$

$$(K_{50})_{Lungs,PACE} = (K_{50})_{Lungs,Au} \times 10^{u_{21}} \tag{10}$$

$$(K_{50})_{Kidneys,PACE} = (K_{50})_{Kidneys,Au} \times 10^{u_{21}} \tag{11}$$

$$(K_{50})_{Spleen,PACE} = (K_{50})_{Spleen,Au} \times 10^{u_{21}} \tag{12}$$

$$PA_{Lungs,PACE} = PA_{Lungs,Au} \times 10^{u_{51}} \tag{13}$$

$$PA_{Bone,PACE} = PA_{Bone,Au} \times 10^{u_{51}} \tag{14}$$

$$PA_{Spleen,PACE} = PA_{Spleen,Au} \times 10^{u_{51}} \tag{15}$$

$$PA_{Heart,PACE} = PA_{Heart,Au} \times 10^{u_{51}} \tag{16}$$

$$PA_{Body,PACE} = PA_{Body,Au} \times 10^{u_{51}} \tag{17}$$

The probabilistic variable u_{ij} is uniformly distributed from -i to j, and is rounded to the nearest whole number:

$$u_{ij} \sim round\big(U(-i\,,j)\big) \tag{18}$$

With each MCIS iteration, u_{ij} was sampled 15 times, one for each parameter. We refer to the above relations, collectively, as a 'sampling protocol.' The distribution u_{ij} was rounded so as to avoid redundancies in the parameter sets, so that the widest range of parameter combinations would be generated over the course of the serial simulations. The choice of ij was based on the bounds on each parameter used to model PEGylated gold NPs; for example, PA values for gold NPs (including 13nm and 100nm diameter NPs) were at most one order of magnitude larger or 5 orders of magnitude smaller than the values used to model the 100nm diameter gold NPs. The one exception are the K_{50} values for the phagocytic organs, which were only on the order of tens of hours in the source material. We opted to include the possibility that K_{50} is on the order of minutes or hundreds of hours.

As suggested by equations 1-6, The 15 parameters estimated above do not constitute the full set of parameters required to run the model. The phagocytic release constants K_{rel} for all phagocytic organs and PA_{Liver} comprise eight additional parameters that needed to be estimated. However, importantly, these parameters can be estimated via correlation with the parameters already estimated above. These correlations follow directly from the steady-state conditions (subscript 'ss') of equations 2-6 (represented, respectively, as equations 7-11):

$$0 = PA\left(\frac{(M_{vasc})_{ss}}{V_{vasc}} - \frac{1}{P}\frac{(M_{extra})_{ss}}{V_{extra}}\right) - (R_{elim})_{ss} \tag{19}$$

$$0 = (R_{up})_{ss} - (R_{rel})_{ss} \tag{20}$$

$$(R_{up})_{ss} = K_{max}(M_{vasc})_{ss} \tag{21}$$

$$(R_{rel})_{ss} = K_{rel}(M_{phago})_{ss} \tag{22}$$

$$(R_{elim})_{ss} = K_{elim}\frac{(M_{extra})_{ss}}{V_{extra}} \tag{23}$$

Where equation 9 is based on equation 4, with $t \rightarrow \infty$. Although we do not have the data to support predictions of the steady-state mass of NPs in any of the model compartments, we can assume that they are finite and nonzero. Indeed, it is possible that the steady-state values are zero, as

the PACE-PEG NPs are eliminated from the mouse over time. This is why we use equations 7-11 to draw correlations between the parameters in a probabilistic manner, not to assume that we know the deterministic relationship between them. From equations 7-11, the following correlations emerge:

$$PA_{Liver} \approx CK_{Bile} \tag{24}$$

$$K_{rel} \approx CK_{max} \tag{25}$$

For C a positive, unknown constant. Lastly, we may estimate K_{max} for the non-liver phagocytic organs by assuming that:

$$(K_{max})_{non-Liver} \approx C(K_{max})_{Liver} \tag{26}$$

The above correlations translate to the following parameter sampling protocols in MCIS:

$$(K_{rel})_{Liver,PACE} = (K_{max})_{Liver,PACE} \times 10^{u_{30}} \tag{27}$$

$$(K_{rel})_{Lungs,PACE} = (K_{max})_{Lungs,PACE} \times 10^{u_{33}} \tag{28}$$

$$(K_{rel})_{Spleen,PACE} = (K_{max})_{Spleen,PACE} \times 10^{u_{33}} \tag{29}$$

$$(K_{rel})_{Kidneys,PACE} = (K_{max})_{Kidneys,PACE} \times 10^{u_{33}} \tag{30}$$

$$(PA_{Liver})_{PACE} = (K_{bile})_{PACE} \times 10^{u_{33}} \tag{31}$$

Note that, unlike the parameter sampling protocol for the parameters estimated by rescaling the gold NP parameters, these parameters are dependent on those 15 parameters initially estimated above (subscript PACE on both sides of each equivalence). Because we cannot ascertain the deterministic relationship between these 'correlated' parameters and the 'estimated' parameters with which they are correlated, we chose i=3 and j=3, to reduce any potential bias in estimating these parameters. The exception to this rule is $(K_{rel})_{Liver}$, which we assume to be equal in order to or less than $(K_{max})_{Liver}$, as this would be required to accumulate NPs in the liver to the point where the ratio of NPs accumulated in the non-liver organs and the liver could possibly be as low as seen experimentally.

The model was run 10^4 times, sampling from u_{ij} to produce 10^4 parameter sets (including the 15 parameters that were estimated by rescaling the gold NP parameters, and the 5 parameters estimated as multiples of the first 15). Each parameter set was used to generate a model time course of NP concentration in the blood and was used to compute endpoint biodistribution for each organ, normalized to the liver. The simulation runs that produced results that lay within the 99% confidence interval of the observed data were identified and the corresponding parameter sets were used to estimate the scaling factors required to generate the biodistribution results seen experimentally. These scaling factors were estimated by averaging the scales (the values of u_{ij}) across the simulation runs whose results were consistent with the data (lying within the 99% confidence interval). If the average of u_{ij} across these 'successful' simulation runs is denoted u_avg, we denote these constant scaling factors as $\sigma_u = 10^{u\_avg}$, recognizing that each of the twenty estimated parameters have their own unique scaling factor, numbered 1-20.

In the second MCIS step, we performed another 10^4 simulation runs, this time sampling from a continuous (unrounded) uniform distribution, v_i:

$$(K_{max})_{Liver,PACE} = (\sigma_u)_1 \times 10^{v_1} \tag{32}$$

$$(K_{max})_{Lungs,PACE} = (\sigma_u)_2 \times 10^{v_1} \tag{33}$$

$$(K_{max})_{Kidneys,PACE} = (\sigma_u)_3 \times 10^{v_1} \tag{34}$$

$$(K_{max})_{Spleen,PACE} = (\sigma_u)_4 \times 10^{v_1} \tag{35}$$

$$(K_{bile})_{PACE} = (K_{bile})_{Au} \times (\sigma_u)_5 \times 10^{v_1} \tag{36}$$

$$(n_H)_{PACE} = (n_H)_{Au} \times (\sigma_u)_6 \times 10^{v_1} \tag{37}$$

$$(K_{50})_{Liver,PACE} = (K_{50})_{Liver,Au} \times (\sigma_u)_7 \times 10^{v_1} \tag{38}$$

$$(K_{50})_{Lungs,PACE} = (K_{50})_{Lungs,Au} \times (\sigma_u)_8 \times 10^{v_1} \tag{39}$$

$$(K_{50})_{Kidneys,PACE} = (K_{50})_{Kidneys,Au} \times (\sigma_u)_9 \times 10^{v_1} \tag{40}$$

$$(K_{50})_{Spleen,PACE} = (K_{50})_{Spleen,Au} \times (\sigma_u)_{10} \times 10^{v_1} \tag{41}$$

$$PA_{Lungs,PACE} = PA_{Lungs,Au} \times (\sigma_u)_{11} \times 10^{v_1} \tag{42}$$

$$PA_{Bone,PACE} = PA_{Bone,Au} \times (\sigma_u)_{12} \times 10^{v_1} \tag{43}$$

$$PA_{Spleen,PACE} = PA_{Spleen,Au} \times (\sigma_u)_{13} \times 10^{v_1} \tag{44}$$

$$PA_{Heart,PACE} = PA_{Heart,Au} \times (\sigma_u)_{14} \times 10^{v_1} \tag{45}$$

$$PA_{Body,PACE} = PA_{Body,Au} \times (\sigma_u)_{15} \times 10^{v_1} \tag{46}$$

$$(K_{rel})_{Liver,PACE} = (K_{max})_{Liver,PACE} \times (\sigma_u)_{16} \times 10^{v_1} \tag{47}$$

$$(K_{rel})_{Lungs,PACE} = (K_{max})_{Lungs,PACE} \times (\sigma_u)_{17} \times 10^{v_1} \tag{48}$$

$$(K_{rel})_{Spleen,PACE} = (K_{max})_{Spleen,PACE} \times (\sigma_u)_{18} \times 10^{v_1} \tag{49}$$

$$(K_{rel})_{Kidneys,PACE} = (K_{max})_{Kidneys,PACE} \times (\sigma_u)_{19} \times 10^{v_1} \tag{50}$$

$$(PA_{Liver})_{PACE} = (K_{bile})_{PACE} \times (\sigma_u)_{20} \times 10^{v_1} \tag{51}$$

For

$$v_i \sim U(-i\,, i) \tag{52}$$

The choice of i = 1 was based on the assumption that the scaling factors σ_u estimated in the first MCIS step were up to one order of magnitude in error, as a result of losing information when the values of u_{ij} were averaged to obtain u_avg for each parameter.

Lastly, we performed a third round of MCIS, sampling from normal distributions, with updated scaling factors σ_{uv} to reflect the scaling performed in the previous iteration:

$$(K_{max})_{Liver,PACE} = (\sigma_{uv})_1 \times 10^{w} \tag{53}$$

$$(K_{max})_{Lungs,PACE} = (\sigma_{uv})_2 \times 10^w \tag{54}$$

$$(K_{max})_{Kidneys,PACE} = (\sigma_{uv})_3 \times 10^w \tag{55}$$

$$(K_{max})_{Spleen,PACE} = (\sigma_{uv})_4 \times 10^w \tag{56}$$

$$(K_{bile})_{PACE} = (K_{bile})_{Au} \times (\sigma_{uv})_5 \times 10^w \tag{57}$$

$$(n_H)_{PACE} = (n_H)_{Au} \times (\sigma_{uv})_6 \times 10^w \tag{58}$$

$$(K_{50})_{Liver,PACE} = (K_{50})_{Liver,Au} \times (\sigma_{uv})_7 \times 10^w \tag{59}$$

$$(K_{50})_{Lungs,PACE} = (K_{50})_{Lungs,Au} \times (\sigma_{uv})_8 \times 10^w \tag{60}$$

$$(K_{50})_{Kidneys,PACE} = (K_{50})_{Kidneys,Au} \times (\sigma_{uv})_9 \times 10^w \tag{61}$$

$$(K_{50})_{Spleen,PACE} = (K_{50})_{Spleen,Au} \times (\sigma_{uv})_{10} \times 10^w \tag{62}$$

$$PA_{Lungs,PACE} = PA_{Lungs,Au} \times (\sigma_{uv})_{11} \times 10^w \tag{63}$$

$$PA_{Bone,PACE} = PA_{Bone,Au} \times (\sigma_{uv})_{12} \times 10^w \tag{64}$$

$$PA_{Spleen,PACE} = PA_{Spleen,Au} \times (\sigma_{uv})_{13} \times 10^w \tag{65}$$

$$PA_{Heart,PACE} = PA_{Heart,Au} \times (\sigma_{uv})_{14} \times 10^w \tag{66}$$

$$PA_{Body,PACE} = PA_{Body,Au} \times (\sigma_{uv})_{15} \times 10^w \tag{67}$$

$$(K_{rel})_{Liver,PACE} = (K_{max})_{Liver,PACE} \times (\sigma_{uv})_{16} \times 10^w \tag{68}$$

$$(K_{rel})_{Lungs,PACE} = (K_{max})_{Lungs,PACE} \times (\sigma_{uv})_{17} \times 10^w \tag{69}$$

$$(K_{rel})_{Spleen,PACE} = (K_{max})_{Spleen,PACE} \times (\sigma_{uv})_{18} \times 10^w \tag{70}$$

$$(K_{rel})_{Kidneys,PACE} = (K_{max})_{Kidneys,PACE} \times (\sigma_{uv})_{19} \times 10^w \tag{71}$$

$$(PA_{Liver})_{PACE} = (K_{bile})_{PACE} \times (\sigma_{uv})_{20} \times 10^w \tag{72}$$

For

$$w \sim N(0, 0.2) \tag{73}$$

We ran the model 10^4 times, sampling from w, to generate the final distribution for each parameter. The parameter sets corresponding to those simulation runs whose results lay within the 99% confidence interval of the experimental data were aggregated and fit to gamma distributions. The choice of these distributions was based on visual inspection of the distributions of parameter values that produced model results that satisfied the experimental data, by performing a QQplot with different distribution fits. Thus, rather than estimating these parameters as single values, we instead assumed the parameter values were distributed probabilistically, such that our model predictions (absolute NP concentrations in the blood and tissue compartments) could be represented as a mean and standard deviation (Figure 4). Importantly, only the initial 15 estimated parameters - K_{max} and K_{50} for each of the phagocytic organs (lungs, kidneys, liver and spleen), the Hill coefficient n_H, K_{bile}, and PA for the non-liver organs, excluding the kidney and including

the ‘body’ compartment – were fit to distributions. The remaining 5 parameters, calculated based on correlations with the first 15, were scaled as in the first MCIS process:

$$(K_{max})_{Liver,PACE} \sim (\sigma_{uv})_1 \times \Gamma(\alpha_1, \beta_1) \quad (74)$$

$$(K_{max})_{Lungs,PACE} \sim (\sigma_{uv})_2 \times \Gamma(\alpha_2, \beta_2) \quad (75)$$

$$(K_{max})_{Kidneys,PACE} \sim (\sigma_{uv})_3 \times \Gamma(\alpha_3, \beta_3) \quad (76)$$

$$(K_{max})_{Spleen,PACE} \sim (\sigma_{uv})_4 \times \Gamma(\alpha_4, \beta_4) \quad (77)$$

$$(K_{bile})_{PACE} \sim (K_{bile})_{Au} \times (\sigma_{uv})_5 \times \Gamma(\alpha_5, \beta_5) \quad (78)$$

$$(n_H)_{PACE} \sim (n_H)_{Au} \times (\sigma_{uv})_6 \times \Gamma(\alpha_6, \beta_6) \quad (79)$$

$$(K_{50})_{Liver,PACE} \sim (K_{50})_{Liver,Au} \times (\sigma_{uv})_7 \times \Gamma(\alpha_7, \beta_7) \quad (80)$$

$$(K_{50})_{Lungs,PACE} \sim (K_{50})_{Lungs,Au} \times (\sigma_{uv})_8 \times \Gamma(\alpha_8, \beta_8) \quad (81)$$

$$(K_{50})_{Kidneys,PACE} \sim (K_{50})_{Kidneys,Au} \times (\sigma_{uv})_9 \times \Gamma(\alpha_9, \beta_9) \quad (82)$$

$$(K_{50})_{Spleen,PACE} \sim (K_{50})_{Spleen,Au} \times (\sigma_{uv})_{10} \times \Gamma(\alpha_{10}, \beta_{10}) \quad (83)$$

$$PA_{Lungs,PACE} \sim PA_{Lungs,Au} \times (\sigma_{uv})_{11} \times \Gamma(\alpha_{11}, \beta_{11}) \quad (84)$$

$$PA_{Bone,PACE} \sim PA_{Bone,Au} \times (\sigma_{uv})_{12} \times \Gamma(\alpha_{12}, \beta_{12}) \quad (85)$$

$$PA_{Spleen,PACE} \sim PA_{Spleen,Au} \times (\sigma_{uv})_{13} \times \Gamma(\alpha_{13}, \beta_{13}) \quad (86)$$

$$PA_{Heart,PACE} \sim PA_{Heart,Au} \times (\sigma_{uv})_{14} \times \Gamma(\alpha_{14}, \beta_{14}) \quad (87)$$

$$PA_{Body,PACE} \sim PA_{Body,Au} \times (\sigma_{uv})_{15} \times \Gamma(\alpha_{15}, \beta_{15}) \quad (88)$$

$$(K_{rel})_{Liver,PACE} = (K_{max})_{Liver,PACE} \times (\sigma_{uv})_{16} \quad (89)$$

$$(K_{rel})_{Lungs,PACE} = (K_{max})_{Lungs,PACE} \times (\sigma_{uv})_{17} \quad (90)$$

$$(K_{rel})_{Spleen,PACE} = (K_{max})_{Spleen,PACE} \times (\sigma_{uv})_{18} \quad (91)$$

$$(K_{rel})_{Kidneys,PACE} = (K_{max})_{Kidneys,PACE} \times (\sigma_{uv})_{19} \quad (92)$$

$$(PA_{Liver})_{PACE} = (K_{bile})_{PACE} \times (\sigma_{uv})_{20} \quad (93)$$

Where Γ denotes the gamma distribution with shape and rate parameters α and β, respectively. Note that the first 15 parameters are distributed probabilistically (denoted by the ~ operator) while the last 5 parameters are calculated based on the first 13 (denoted by the = operator).

After two rounds of re-parameterizing the model using MCIS, inaccuracies still occur. Namely, by assuming the bounds on the possible values of the parameters (i and j in u_{ij}), there is no guarantee that the model will actually fit the experimental data. For instance, due to the bounds set on the maximum liver phagocytosis rate, without manual intervention the amount of NPs estimated for the non-liver organs was far too high. As a result, the parameters were further modified manually to fit the biodistribution data, as previously described. This was performed by multiplying the parameters by different constants, until the model fit the biodistribution data (Supplementary

Figure 3a). These constants, reflected in the code that is freely accessible (at github.com) can be incorporated into another round of MCIS to re-parameterize the model, but for the intents and purposes of this study the constants are kept and reported in the code only. The final distributions for the model parameters are tabulated in Supplementary Table 3, for each parameter in each organ compartment.

Supplementary References

1. Lin, Z., Monteiro-Riviere, N. A. & Riviere, J. E. A physiologically based pharmacokinetic model for polyethylene glycol-coated gold nanoparticles of different sizes in adult mice. *Nanotoxicology* **10**, 162-172 (2016). https://doi.org:10.3109/17435390.2015.1027314

2. Marenzana, M. & Arnett, T. R. The Key Role of the Blood Supply to Bone. *Bone Res* **1**, 203-215 (2013). https://doi.org:10.4248/BR201303001

3. Davies, B. & Morris, T. Physiological parameters in laboratory animals and humans. *Pharm Res* **10**, 1093-1095 (1993). https://doi.org:10.1023/a:1018943613122

4. Nombela-Arrieta, C. & Manz, M. G. Quantification and three-dimensional microanatomical organization of the bone marrow. *Blood Adv* **1**, 407-416 (2017). https://doi.org:10.1182/bloodadvances.2016003194

5. Covarrubias, R. *et al.* Optimized protocols for isolation, fixation, and flow cytometric characterization of leukocytes in ischemic hearts. *Am J Physiol Heart Circ Physiol* **317**, H658-H666 (2019). https://doi.org:10.1152/ajpheart.00137.2019

6. Falconer, D. S., Gauld, I. K. & Roberts, R. C. Cell numbers and cell sizes in organs of mice selected for large and small body size. *Genet Res* **31**, 287-301 (1978). https://doi.org:10.1017/s0016672300018061

7. Albert, C. *et al.* Monobody adapter for functional antibody display on nanoparticles for adaptable targeted delivery applications. *Nat Commun* **13**, 5998 (2022). https://doi.org:10.1038/s41467-022-33490-8

8. Cui, J. *et al.* Poly(amine-co-ester) nanoparticles for effective Nogo-B knockdown in the liver. *J Control Release* **304**, 259-267 (2019). https://doi.org:10.1016/j.jconrel.2019.04.044

9. Tokdar, S. T. & Kass, R. E. Importance Sampling: A Review. *Wiley Interdisciplinary Reviews: Computational Statistics* **2.1**, 54-60 (2010).
