# Supplementary material for: Enhancing in vivo cell and tissue targeting by modulation of polymer nanoparticles and macrophage decoys
Source: Nat Commun. 2024 May 18;15:4247. doi: 10.1038/s41467-024-48442-7 (PMC11102454; doi:10.1038/s41467-024-48442-7)
Supplement: Supplementary file 2 — Reporting Summary [file 41467_2024_48442_MOESM2_ESM.pdf]

nature portfolio

Corresponding author(s): Alexandra Piotrowski-Daspit, Laura Bracaglia, and W. Mark Saltzman

Last updated by author(s): April 22, 2024

Reporting Summary

Nature Portfolio wishes to improve the reproducibility of the work that we publish. This form provides structure for consistency and transparency in reporting. For further information on Nature Portfolio policies, see our Editorial Policies and the Editorial Policy Checklist.

Statistics

For all statistical analyses, confirm that the following items are present in the figure legend, table legend, main text, or Methods section.

n/a	Confirmed	
☐	☒	The exact sample size (*n*) for each experimental group/condition, given as a discrete number and unit of measurement
☐	☒	A statement on whether measurements were taken from distinct samples or whether the same sample was measured repeatedly
☐	☒	The statistical test(s) used AND whether they are one- or two-sided<br>*Only common tests should be described solely by name; describe more complex techniques in the Methods section.*
☒	☐	A description of all covariates tested
☐	☒	A description of any assumptions or corrections, such as tests of normality and adjustment for multiple comparisons
☐	☒	A full description of the statistical parameters including central tendency (e.g. means) or other basic estimates (e.g. regression coefficient) AND variation (e.g. standard deviation) or associated estimates of uncertainty (e.g. confidence intervals)
☐	☒	For null hypothesis testing, the test statistic (e.g. *F*, *t*, *r*) with confidence intervals, effect sizes, degrees of freedom and *P* value noted<br>*Give P values as exact values whenever suitable.*
☐	☒	For Bayesian analysis, information on the choice of priors and Markov chain Monte Carlo settings
☒	☐	For hierarchical and complex designs, identification of the appropriate level for tests and full reporting of outcomes
☒	☐	Estimates of effect sizes (e.g. Cohen's *d*, Pearson's *r*), indicating how they were calculated

Our web collection on statistics for biologists contains articles on many of the points above.

Software and code

Policy information about availability of computer code

Data collection	Flow cytometry data was collected using BD FACSDiva (BD Biosciences). Fluorescence and bright field images were collected using EVOS FL Auto 2 Software (ThermoFisher Scientific). IVIS images were collected with Living Image 4.7.3 (by Perkin Elmer). Nanoparticle physical characteristics were measured using Zetasizer Advance (Malvern Panalytical). PCR data was collected using StepOne Software version 2.3.
Data analysis	Flow cytometry data was analyzed using FlowJo v10. Fluorescence images were analyzed using ImageJ 1.5.2 (NIH) and a custom MATLAB program. IVIS images were analyzed using Living Image 4.7.3 (by Perkin Elmer). Mathematic and statistical analysis were performed in Excel (Microsoft) and Graphpad Prism (version 10.2.2). With respect to the PBPK model, data analysis and mathematical modeling was performed in R, with PBPK model figures visualized in MATLAB. The model source code and data used to parameterize the model are available at: omrichfield/PACE-PBPK-Monte-Carlo__public: Public Physiologically Based Pharmacokinetic Model of polyamine coester nanoparticles (github.com, DOI: 10.5281/zenodo.10999309).

For manuscripts utilizing custom algorithms or software that are central to the research but not yet described in published literature, software must be made available to editors and reviewers. We strongly encourage code deposition in a community repository (e.g. GitHub). See the Nature Portfolio guidelines for submitting code & software for further information.

Data

Policy information about availability of data

All manuscripts must include a data availability statement. This statement should provide the following information, where applicable:

- Accession codes, unique identifiers, or web links for publicly available datasets
- A description of any restrictions on data availability
- For clinical datasets or third party data, please ensure that the statement adheres to our policy

The data generated to support the findings in this study are contained in the article and the Supplementary Information. Raw images (blood quantification and IVIS) and FCS files can be provided upon request. All raw data for model development were generated in this study and can be found at: omrichfield/PACE-PBPK-Monte-Carlo__public: Public Physiologically Based Pharmacokinetic Model of polyamine coester nanoparticles (github.com, DOI: 10.5281/zenodo.10999309).

Research involving human participants, their data, or biological material

Policy information about studies with human participants or human data. See also policy information about sex, gender (identity/presentation), and sexual orientation and race, ethnicity and racism.

Reporting on sex and gender	This information has not been collected.
Reporting on race, ethnicity, or other socially relevant groupings	This information has not been collected.
Population characteristics	This information has not been collected.
Recruitment	Participants were recruited at the Yale School of Medicine. Blood samples from healthy donors who provided informed consent were collected at the Yale School of Medicine. Participants were not compensated for their enrollment, limiting self-selection bias.
Ethics oversight	All collections were performed under protocols approved by the Yale University Institutional Review Board (IRB).

Note that full information on the approval of the study protocol must also be provided in the manuscript.

Field-specific reporting

Please select the one below that is the best fit for your research. If you are not sure, read the appropriate sections before making your selection.

[x] Life sciences [ ] Behavioural & social sciences [ ] Ecological, evolutionary & environmental sciences

For a reference copy of the document with all sections, see nature.com/documents/nr-reporting-summary-flat.pdf

Life sciences study design

All studies must disclose on these points even when the disclosure is negative.

Sample size	Animal experiments were conducted with at least n=3 animals, as determined by a power analysis using a T test and previously published data from Bracaglia & Piotrowski-Daspit, 2019 PNAS, which reports detectable differences between various NP pharmacokinetics and follows the same procedure as reported here (alpha = 0.05, observed difference in NP concentration at 4 hours is 95mg/mL, standard deviation of 10, and power of 0.9). Human blood experiments were likewise conducted with at least n=3 blood samples. While no sample size calculation was performed prior to the study, previously published data using the monobody and antibody-mediated targeting technology described here from Albert and Bracaglia, 2022, Nature Communications, showed highly significant differences between targeted and isotype controls.
Data exclusions	No data were excluded from the analyses.
Replication	All attempts at replication were successful and each experiment was performed independently at least three times.
Randomization	Samples/organisms/participants were randomly allocated into experimental groups.
Blinding	All studies were partially blinded. Investigators knew the identity of the NPs or other treatment administered for logistical purposes, but were blind to any animal or sample specifics that may determine or enhance results.

Reporting for specific materials, systems and methods

We require information from authors about some types of materials, experimental systems and methods used in many studies. Here, indicate whether each material, system or method listed is relevant to your study. If you are not sure if a list item applies to your research, read the appropriate section before selecting a response.

Materials & experimental systems

n/a	Involved in the study	
	x	Antibodies
x		Eukaryotic cell lines
x		Palaeontology and archaeology
	x	Animals and other organisms
x		Clinical data
x		Dual use research of concern
x		Plants

Methods

n/a	Involved in the study	
x		ChIP-seq
	x	Flow cytometry
x		MRI-based neuroimaging

Antibodies

Antibodies used	Monoclonal antibodies for flow cytometry were purchased from ThermoFisher Scientific (FITC anti-mouse CD45 (30-F11, Catalog # 11-0451-82, Lot # 2306900), PE anti-mouse CD31 (390, Catalog # 12-0311-82, Lot # 2114546), anti-human CD4 (9H5A8, Catalog # MA5-15774, Lot # 3558711), anti-rat CD4 (W3/25, Catalog # MA5-17387, Lot # XG3637671), PE anti-rat CD3 (G4.18, Catalog # 12-0030-82, Lot # 2372960)) and Biolegend (PE anti-mouse F4/80 (BM8, Catalog # 123110, Lot # B340064), PE anti-mouse CD326 (G8.8, Catalog # 118206, Lot # B297468 and B355934), PE anti-human CD3 (UCHT1, Catalog # 980008, Lot # B342969), FITC anti-human CD8a (RPA-T8, Catalog # 301060, Lot #B331658), FITC anti-rat CD8a (OX-8, Catalog # 201703, Lot # B353364)). Anti-mouse RBC monoclonal antibody (34-3C) was purchased from Hycult Biotech (Catalog # HM1120-100UG).
Validation	Each of the antibodies reported have been tested/validated by the vendor to bind to the intended antigen for the appropriate application. These statements are provided on the vendor product pages:<br>FITC anti-mouse CD45 (30-F11, Catalog # 11-0451-82): https://www.thermofisher.com/antibody/product/CD45-Antibody-clone-30-F11-Monoclonal/11-0451-82<br>PE anti-mouse CD31 (390, Catalog # 12-0311-82): https://www.thermofisher.com/antibody/product/CD31-PECAM-1-Antibody-clone-390-Monoclonal/12-0311-82<br>anti-human CD4 (9H5A8, Catalog # MA5-15774): https://www.thermofisher.com/antibody/product/CD4-Antibody-clone-9H5A8-Monoclonal/MA5-15774<br>anti-rat CD4 (W3/25, Catalog # MA5-17387): https://www.thermofisher.com/antibody/product/CD4-Antibody-clone-W3-25-Monoclonal/MA5-17387<br>PE anti-rat CD3 (G4.18, Catalog # 12-0030-82): https://www.thermofisher.com/antibody/product/CD3-Antibody-clone-eBioG4-18-G4-18-Monoclonal/12-0030-82<br>PE anti-mouse F4/80 (BM8, Catalog # 123110): https://www.biolegend.com/en-us/products/pe-anti-mouse-f4-80-antibody-4068<br>PE anti-mouse CD326 (G8.8, Catalog # 118206): https://www.biolegend.com/en-us/products/pe-anti-mouse-cd326-ep-cam-antibody-4726<br>PE anti-human CD3 (UCHT1, Catalog # 980008): https://www.biolegend.com/en-us/products/pe-anti-human-cd3-antibody-17523<br>FITC anti-human CD8a (RPA-T8, Catalog # 301060): https://www.biolegend.com/en-us/products/fitc-anti-human-cd8a-antibody-834<br>FITC anti-rat CD8a (OX-8, Catalog # 201703): https://www.biolegend.com/en-us/products/fitc-anti-rat-cd8a-antibody-2388<br>Anti-mouse RBC monoclonal antibody (34-3C, Catalog # HM1120-100UG): https://www.hycultbiotech.com/product/rbc-mouse-mab-34-3c/

Animals and other research organisms

Policy information about studies involving animals; ARRIVE guidelines recommended for reporting animal research, and Sex and Gender in Research

Laboratory animals	BALB/c mice (4-6 weeks), C57BL/6 mice (4-6 weeks), C57BL/6 mice homozygous for the F508del mutation (3-6 months), Sprague Dawley rats (3-5 weeks)
Wild animals	This study did not involve wild animals.
Reporting on sex	Animals of both sexes were randomly assigned to experimental groups for mice studies. Female Sprague Dawley rats were used for in vivo CD4 targeting experiments. Data have not been disaggregated for sex/sex-based analyses were not performed based on previously published data from Bracaglia & Piotrowski-Daspit, 2019 PNAS, which showed no significant differences in polymeric NP biodistribution based on sex.
Field-collected samples	This study did not involve samples collected from the field.
Ethics oversight	All animal procedures were performed in accordance with the guidelines and policies of the Yale Animal Resources Center (YARC) and approved by the Institutional Animal Care and Use Committee (IACUC) of Yale University (IACUC Protocol Number 2020-11228).

Note that full information on the approval of the study protocol must also be provided in the manuscript.

Flow Cytometry

Plots

Confirm that:

- [x] The axis labels state the marker and fluorochrome used (e.g. CD4-FITC).
- [x] The axis scales are clearly visible. Include numbers along axes only for bottom left plot of group (a 'group' is an analysis of identical markers).
- [x] All plots are contour plots with outliers or pseudocolor plots.
- [x] A numerical value for number of cells or percentage (with statistics) is provided.

Methodology

Sample preparation

As described in the methods section: For tissue homogenization, hearts, spleens, and kidneys were homogenized into a single-cell suspension through a 70 um cell strainer with RPMI 1640 culture medium containing 10% FBS, washed once with DPBS by centrifugation, and resuspended in DPBS containing 2% bovine serum albumin (BSA) and Hoescht 33342 (5 ug/mL). To extract cells from the bone marrow, both ends of one femur were cut, and the femur was flushed with RPMI 1640 culture medium containing 10% FBS using a 25G needle and syringe. Bone marrow cells were then washed once with PBS by centrifugation and resuspended in DPBS containing 2% BSA and 5 ug/mL Hoescht. Livers and lungs were processed further to determine PACE NP distribution in individual cell populations. Livers and lungs were first chopped manually and then digested using a solution of DNase (1 mg/mL) and Collagenase Type I (5 mg/mL) in HBSS at 37°C for 30 minutes on an orbital shaker. The resulting liver cell suspension was then filtered through a 70 um cell strainer and rinsed twice with HBSS containing 4.8% BSA and 2mM EDTA. Cells were collected by centrifugation at 330 x g for 5 minutes at 4°C. The cell pellet was then suspended in 2 mL of 1X RBC Lysis Buffer (Invitrogen) for 2 minutes, rinsed with 5 mL DPBS containing 2% BSA, and then collected again by centrifugation as described. Following collagenase and DNase digestion, the resulting lung cell suspension was further separated with shearing through an 18G needle, filtered through a 70 um cell strainer, and rinsed twice with DPBS containing 0.5% BSA. Cells were collected by centrifugation at 330 x g for 5 minutes at 4°C. The cell pellet was then suspended in 2 mL of 1X RBC Lysis Buffer for 2 minutes, rinsed with 5 mL of DPBS containing 2% BSA, and then collected again by centrifugation as described. Cell suspensions from liver and lung were stained for cell specific markers using fluorescently labeled antibodies. Diluted cell suspensions containing 1 mil cells were stained with 5 uL of antibody for 1 hour on ice. Unbound antibodies were rinsed off using 1 mL of DPBS containing 2% BSA, and cells were collected by centrifugation. Stained cells were then suspended in DPBS containing 2% BSA and 5 ug/mL Hoescht.

For CD4 targeting analysis of nucleated cells in human or rat blood: Nucleated cells were isolated from the peripheral blood samples and processed for flow cytometry. Briefly, 200-300 uL of blood per sample was mixed with 50 uL of heparin prior to centrifugation for 3 min at 1800 rpm and 4°C. The supernatant was aspirated, and the remaining pellet was incubated with 1 mL of ACK lysis buffer and incubated for 3 min prior to centrifugation at 1800 rpm and 4°C (repeated twice). The pelleted cells were then resuspended in cold 1X DPBS and stained with antibodies for anti-human CD3 (clone UCHT1) and anti-human CD8a (clone RPA-T8). NP uptake in isolated and stained nucleated cells from peripheral blood was then analyzed by flow cytometry (BD LSRII).

Instrument

BD LSRII

Software

BD FACSDiva was used to collect flow cytometry data and FlowJo was used to analyze the data.

Cell population abundance

Each tissue type for each animal represented was sampled to obtain at least 20,000 nucleated, single cells. For lung and liver tissues, cells were further sorted into CD31+ cells, F4/80+ cells, CD45+ cells, and EpCAM+ cells. These populations consistently contained at least 3000 cells per sample, and represented 17% (CD31+), 17% (F4/80+), 12% (CD45+), 11% (EpCAM +) on average of the nucleated, single cell population.

Gating strategy

Cells were first gated for nucleated cells using a hoechst stain. Nucleated cells were then selected for single cells using a forward scatter height by forward scatter area selection. From there, bulk cells were evaluated for NP signal using the fluorescent dye contained within the NP (DiD -APC channel), and compared to cells from animals with out NPs. Bulk cells were also sorted into specific cell populations, using fluorescent antibody markers, and gated based on unstained cells from the same population.

- [x] Tick this box to confirm that a figure exemplifying the gating strategy is provided in the Supplementary Information.
